# Supplementary material for: Extended or Continuous Infusion of Carbapenems in Children with Severe Infections: A Systematic Review and Narrative Synthesis
Source: Antibiotics (Basel). 2021 Sep 9;10(9):1088. doi: 10.3390/antibiotics10091088 (PMC8470113; doi:10.3390/antibiotics10091088)
Supplement: Supplementary file 1 [file antibiotics-10-01088-s001.zip › antibiotics-1353414-supplementary.pdf]

Supplementary Materials

Review

Extended or Continuous Infusion of Carbapenems in Children with Severe Infections: A Systematic Review and Narrative Synthesis

Pengxiang Zhou [1,2,†], Yahui Zhang [1,3,†], Zhenhuan Wang [3,4], Yingqiu Ying [1], Yan Xing [5], Xiaomei Tong [5,*] and Suodi Zhai [1,2,*]

Table S1. PRISMA checklist

Section and Topic	Item #	Checklist item	Location where item is reported
**TITLE**			
Title	1	Identify the report as a systematic review.	P1
**ABSTRACT**			
Abstract	2	See the PRISMA 2020 for Abstract's checklist.	P1
**INTRODUCTION**			
Rationale	3	Describe the rationale for the review in the context of existing knowledge.	P2
Objectives	4	Provide an explicit statement of the objective(s) or question(s) the review addresses.	P2
**METHODS**			
Eligibility criteria	5	Specify the inclusion and exclusion criteria for the review and how studies were grouped for the syntheses.	P13
Information sources	6	Specify all databases, registers, websites, organisations, reference lists and other sources searched or consulted to identify studies. Specify the date when each source was last searched or consulted.	P13
Search	7	Present the full search strategies for all databases, registers, and websites, including any filters and limits	P13, Table S2

Section and Topic	Item #	Checklist item	Location where item is reported
strategy		used.	
Selection process	8	Specify the methods used to decide whether a study met the inclusion criteria of the review, including how many reviewers screened each record and each report retrieved, whether they worked independently, and if applicable, details of automation tools used in the process.	P13
Data collection process	9	Specify the methods used to collect data from reports, including how many reviewers collected data from each report, whether they worked independently, any processes for obtaining or confirming data from study investigators, and if applicable, details of automation tools used in the process.	P13
Data items	10a	List and define all outcomes for which data were sought. Specify whether all results that were compatible with each outcome domain in each study were sought (e.g. for all measures, time points, analyses), and if not, the methods used to decide which results to collect.	P13
	10b	List and define all other variables for which data were sought (e.g. participant and intervention characteristics, funding sources). Describe any assumptions made about any missing or unclear information.	P13
Study risk of bias assessment	11	Specify the methods used to assess risk of bias in the included studies, including details of the tool(s) used, how many reviewers assessed each study and whether they worked independently, and if applicable, details of automation tools used in the process.	P14
Effect measures	12	Specify for each outcome the effect measure(s) (e.g. risk ratio, mean difference) used in the synthesis or presentation of results.	P13
Synthesis methods	13a	Describe the processes used to decide which studies were eligible for each synthesis (e.g. tabulating the study intervention characteristics and comparing against the planned groups for each synthesis (item #5)).	P14
	13b	Describe any methods required to prepare the data for presentation or synthesis, such as handling of missing summary statistics, or data conversions.	P14
	13c	Describe any methods used to tabulate or visually display results of individual studies and syntheses.	P14
	13d	Describe any methods used to synthesize results and provide a rationale for the choice(s). If meta-analysis was performed, describe the model(s), method(s) to identify the presence and extent of statistical	P14

Section and Topic	Item #	Checklist item	Location where item is reported
		heterogeneity, and software package(s) used.	
	13e	Describe any methods used to explore possible causes of heterogeneity among study results (e.g. subgroup analysis, meta-regression).	P14
	13f	Describe any sensitivity analyses conducted to assess robustness of the synthesized results.	P14
Reporting bias assessment	14	Describe any methods used to assess risk of bias due to missing results in a synthesis (arising from reporting biases).	P14
Certainty assessment	15	Describe any methods used to assess certainty (or confidence) in the body of evidence for an outcome.	NA*
**RESULTS**			
Study selection	16a	Describe the results of the search and selection process, from the number of records identified in the search to the number of studies included in the review, ideally using a flow diagram.	P2, Fig 1
	16b	Cite studies that might appear to meet the inclusion criteria, but which were excluded, and explain why they were excluded.	Fig 1
Study characteristics	17	Cite each included study and present its characteristics.	P3, Tab 1
Risk of bias in studies	18	Present assessments of risk of bias for each included study.	P2, Tab S3
Results of individual studies	19	For all outcomes, present, for each study: (a) summary statistics for each group (where appropriate) and (b) an effect estimates and its precision (e.g. confidence/credible interval), ideally using structured tables or plots.	P3-P6
Results of syntheses	20a	For each synthesis, briefly summarise the characteristics and risk of bias among contributing studies.	P7-P11
	20b	Present results of all statistical syntheses conducted. If meta-analysis was done, present for each the summary estimate and its precision (e.g. confidence/credible interval) and measures of statistical heterogeneity. If comparing groups, describe the direction of the effect.	P7-P11

Section and Topic	Item #	Checklist item	Location where item is reported
	20c	Present results of all investigations of possible causes of heterogeneity among study results.	NA
	20d	Present results of all sensitivity analyses conducted to assess the robustness of the synthesized results.	NA
Reporting biases	21	Present assessments of risk of bias due to missing results (arising from reporting biases) for each synthesis assessed.	P2
Certainty of evidence	22	Present assessments of certainty (or confidence) in the body of evidence for each outcome assessed.	P2
**DISCUSSION**			
Discussion	23a	Provide a general interpretation of the results in the context of other evidence.	P12
	23b	Discuss any limitations of the evidence included in the review.	P13
	23c	Discuss any limitations of the review processes used.	P13
	23d	Discuss implications of the results for practice, policy, and future research.	P12-P13
**OTHER INFORMATION**			
Registration and protocol	24a	Provide registration information for the review, including register name and registration number, or state that the review was not registered.	P13
	24b	Indicate where the review protocol can be accessed, or state that a protocol was not prepared.	P13
	24c	Describe and explain any amendments to information provided at registration or in the protocol.	None
Support	25	Describe sources of financial or non-financial support for the review, and the role of the funders or sponsors in the review.	P14
Competing interests	26	Declare any competing interests of review authors.	P14
Availability of data, code and other	27	Report which of the following are publicly available and where they can be found: template data collection forms; data extracted from included studies; data used for all analyses; analytic code; any other materials used in the review.	P

Section and Topic	Item #	Checklist item	Location where item is reported
materials			

Note: * Grading of recommendations assessment, development and evaluation (GRADE) tool was not appropriate for this review owing to the insufficient data of included controlled studies.

Table S2. Search strategies

Databases	Detailed search strategies
PubMed	#**1** Meropenem[Title/Abstract] OR (Meropenem[MeSH Terms] OR Imipenem[MeSH Terms] OR Imipenem[Title/Abstract] OR Biapenem[Supplementary Concept] OR Biapenem[Title/Abstract] OR Panipenem[Supplementary Concept] OR Panipenem[Title/Abstract] OR Carbapenems[MeSH Terms] OR Carbapenems [Title/Abstract]<br>#**2** Infusions, Intravenous[MeSH Terms] OR Infusion[Title/Abstract] OR intravenous[Title/Abstract] OR parenteral[Title/Abstract] OR drip[Title/Abstract] OR drops[Title/Abstract]<br>#**3** Continuous OR Prolonged OR Extended OR Discontinu* OR intermittent OR interval OR Drug administration schedule[MeSH Terms]<br>**#1 AND #2 AND #3**<br>**Filters:** Child: birth-18 years
Embase	#**1** 'meropenem'/exp OR 'meropenem' OR meropenem.ti,ab OR 'imipenem'/exp OR 'imipenem' OR imipenem.ti,ab OR 'biapenem'/exp OR 'biapenem' OR 'panipenem'/exp OR 'panipenem' OR biapenem.ti,ab OR 'carbapenem'/exp OR 'carbapenem' OR carbapenem.ti,ab<br>#**2** 'intravenous drug administration'/exp OR infusion OR intravenous OR parenteral OR drip OR drops<br>#**3** continuous OR prolonged OR extended OR discontinuous OR intermittent OR interval OR 'dosage schedule comparison' OR 'drug intermittent therapy'/exp<br>**#1 AND #2 AND #3**<br>Filters: [newborn]/lim OR [infant]/lim OR [child]/lim OR [preschool]/lim OR [school]/lim OR [adolescent]/lim
The Cochrane Library	#**1** Meropenem[Title/Abstract/Key words] OR Meropenem[MeSH Terms] OR Imipenem[MeSH Terms] OR Imipenem[Title/Abstract] OR Biapenem[Supplementary Concept] OR Biapenem[Title/Abstract] OR Panipenem[Supplementary Concept] OR Panipenem[Title/Abstract] OR Carbapenems[MeSH Terms] OR Carbapenems [Title/Abstract]<br>#**2** Infusions, Intravenous [Mesh Terms] OR Infusion OR intravenous OR parenteral OR drip OR drips

	**#3** Continuous OR Prolonged OR Extended OR Dsicontinu* or intermittent or interval OR Drug Administration Schedule [MeSH Terms]<br>**#4** Adolescent [MeSH Terms] OR Child [MeSH Terms] OR infant [MeSH Terms] OR nfant* OR neonat* OR newborn OR teen* OR preschool* OR school* OR adolescen* OR toddler* OR pubert* OR minor* OR prematur* OR juvenile OR pediatric* OR paediatric* OR child* OR girl OR boy OR baby OR kid (word variations have been searched)<br>**#1 AND #2 AND #3 AND #4**

Table S3. Results of risk bias assessment included in RCTs

Reference	**Random sequence generation (selection bias)**	**Allocation concealment (selection bias)**	**Blinding of participants and personnel (performance bias)**	**Blinding of outcome assessment (detection bias)**	**Incomplete outcome data (attrition bias)**	**Selective reporting**	**Other bias**
Shabaan 2017	L	L	H	L	L	L	L
Wang 2018	H	U	U	U	L	L	L

Note: U: Unclear risk; L: Low risk; H: High risk
